# Supplementary material for: Demographic variation in symptoms of depression and anxiety across 22 Global Flourishing Study countries
Source: Commun Med (Lond). 2026 Jan 9;6:100. doi: 10.1038/s43856-025-01366-9 (PMC12891731; doi:10.1038/s43856-025-01366-9)
Supplement: Supplementary file 2 — Description of Additional Supplementary Data [file 43856_2025_1366_MOESM2_ESM.docx]

Description of additional supplementary file

File name: Supplementary Data

Description: Demographic Variation in Symptoms of Depression and Anxiety Across 22 Global Flourishing Study Countries
